# COVID-19 AND THE FEAR OF FALLING AMONG OLDER ADULTS IN SINGAPORE: AN ELECTRONIC SURVEY

**DOI:** 10.1093/geroni/igac059.1082

**Published:** 2022-12-20

**Authors:** Wayne Chong, Tharshini Lokanathan, W Quin Yow

**Affiliations:** Nanyang Technological University, Singapore, Singapore, Singapore; Singapore University of Technology & Design, Singapore, Singapore; Singapore University of Technology & Design, Singapore, Singapore

## Abstract

We investigated the predictors of fear of falling in Singapore during the COVID-19 pandemic. Older adults aged 60 to 85 (N=144) participated in an electronic survey that assessed their attitudes toward technology, perceived severity and fear of COVID, frailty, social engagement and falls risk. Hierarchical linear regressions revealed that the fear of falling was first predicted by falls risk, beta = .26, p = .001, F(1, 142) = 10.50, then by resistance, beta = .18, p = .03, F(2, 141) = 7.96. When COVID-19 fear, beta = .14, p < .001, and health conditions, beta = -.22, p = .005, were added together, falls risk became non-significant, beta = .14, p = .083, F(4, 139) = 10.50. The interaction between COVID-19 fear and health conditions was found to add significant variance in the final model, beta = -.31, p < .001, R square = .37, R square change = .09.

